# MiR-125b-5p promotes pemphigus vulgaris pathogenesis by modulating desmosome structures

**DOI:** 10.1042/BSR20260220

**Published:** 2026-07-30

**Authors:** Wenxiu He, Tingning Xiao, Hong Hua, Yixiao Xing, Panpan Shang, Jingya Fu, Li Wu Zheng, Pan Wei

**Affiliations:** 1 Department of Stomatology, Peking Union Medical College Hospital, Chinese Academy of Medical Sciences & Peking Union Medical College, Beijing, PR China; 2 Department of Oral Medicine, Peking University School and Hospital of Stomatology & National Center of Stomatology & National Clinical Research Center for Oral Diseases & National Engineering Laboratory for Digital and Material Technology of Stomatology & Beijing Key Laboratory of Digital Stomatology & Research Center of Engineering and Technology for Computerized Dentistry Ministry of Health & NMPA Key Laboratory for Dental Materials, Beijing, PR China; 3 Division of Oral & Maxillofacial Surgery, Faculty of Dentistry, The University of Hong Kong, Hong Kong SAR, PR China; 4 Department of Stomatology, Zhongshan Hospital, Fudan University, Shanghai, PR China; 5 Department of Dermatology, Peking University First Hospital & National Clinical Research Center for Skin and Immune Diseases & Beijing Key Laboratory of Molecular Diagnosis on Dermatoses & National Medical Products Administration Key Laboratory for Quality Control and Evaluation of Cosmetics, Beijing, PR China; 6 First Clinical Division, Peking University School and Hospital of Stomatology & National Center of Stomatology & National Clinical Research Center for Oral Diseases & National Engineering Laboratory for Digital and Material Technology of Stomatology & Beijing Key Laboratory of Digital Stomatology & Research Center of Engineering and Technology for Computerized Dentistry Ministry of Health & NMPA Key Laboratory for Dental Materials, Beijing, PR China

**Keywords:** desmosomes, miR-125b-5p, P63, pemphigus vulgaris, PERP

## Abstract

Pemphigus vulgaris (PV) is a life-threatening autoimmune bullous disease with an unclear pathogenesis. Our previous study indicated that the expression of miR-125b-5p was significantly elevated in PV patients. MiR-125b-5p has been reported to target P63 mRNA and inhibit the expression of P63, while p53-apoptosis-effector-related-to-PMP-22 (PERP) is a downstream protein regulated by P63. However, the role and underlying molecular mechanisms of miR-125b-5p in PV remain unclear. Here, we explored the roles of miR-125b-5p, P63, and PERP in regulating desmosome integrity and their contribution to PV pathogenesis. The dual-luciferase reporter assay showed that miR-125b-5p inhibited P63 expression by directly binding to the 3′ UTR of P63 mRNA. Mouse tongue tissues were cultured *in vitro* and treated with desmoglein (Dsg) 3-monoclonal antibody (mAb) or with miR-125b-5p overexpression before Dsg3-mAb treatment. Overexpression of miR-125b-5p significantly reduced the mRNA and protein levels of P63 and PERP in Dsg3-mAb-treated tongue tissue. Transmission electron microscopy analysis of the structure of desmosomes revealed that, in tongue tissue with miR-125b-5p overexpression and Dsg3-mAb treatment, the interdesmosal width was significantly increased, the thickness of keratin insertion into the membrane was markedly reduced, and the desmosome structures exhibited evident damage. Together, these findings indicate that miR-125b-5p reduced the expression of both P63 and PERP by targeting P63, thereby contributing to the disruption of desmosome integrity.

## Introduction

Pemphigus is a severe mucocutaneous autoimmune bullous disease, of which pemphigus vulgaris (PV) is the most common subtype, accounting for 65%–90% of cases [1,2]. Oral lesions manifest as the initial symptom in 50%–90% of PV patients and persist in nearly all patients throughout the disease course [3,4]. The autoantibodies in PV predominantly target desmosomal components in the epidermis, particularly desmoglein (Dsg) 3 and Dsg1, leading to mucosal and/or cutaneous blistering [2,5]. The mucocutaneous type of PV predominantly affects mucosa, with minimal or absent cutaneous involvement, where Dsg3 serves as the primary pathogenic autoantibody [2,4]. With the advent of corticosteroids, monoclonal antibodies, and other therapeutic advances, the mortality rate of PV has been significantly reduced; however, it remains 2.4–3.3 times higher than that of the general population [6,7]. To date, the precise pathogenesis of PV remains unclear, which hinders the development of targeted and effective therapies.

MicroRNAs (miRNAs) are endogenous, non-coding, single-stranded small RNA molecules that function as regulators of multiple biological processes and pathways, including apoptosis, cell differentiation, intercellular communication, and immune homeostasis [8]. In autoimmune diseases, miRNAs can serve as biomarkers for clinical monitoring and diagnosis, and they are involved in disease pathogenesis through various pathways, including T cell activation, inflammatory signaling, cytokine production, and cell apoptosis [9–12]. The role of miRNAs in the pathogenesis of PV has not been clearly defined and warrants further investigation.

Our previous study found that miR-125b-5p expression was significantly elevated in the serum of PV patients [13]. MiR-125b-5p is a key miRNA that regulates 5.57% of the human gene transcripts [14]. P63 is a significant regulator of epithelial development and desmosome function [15,16]. A previous study confirmed that miR-125b-5p inhibits P63 expression by targeting its mRNA, suggesting that P63 is the direct target of miR-125b-5p [17]. PERP, a p53-targeted apoptosis effector related to PMP-22, is an adhesion protein involved in desmosome formation and essential for maintaining desmosome integrity and cell adhesion [18]. PERP is a transcriptional target of P63, and its expression depends on P63 [16]. To date, the molecular mechanisms by which miR-125b-5p and its downstream targets, including P63 and PERP, influence PV pathogenesis remain poorly understood. Therefore, the present study aims to investigate the roles of miR-125b-5p, P63, and PERP in regulating desmosome integrity and their contribution to PV pathogenesis.

## Methods

### Mice and ethics statement

Animal experiments performed followed the National Research Council Guide for the Care and Use of Laboratory Animals. All animal procedures were approved by the Ethics Committee of Peking University Health Science Center (LA2022222). Eight-week-old adult BALB/c mice (specific pathogen-free (SPF) grade) were purchased from Beijing Vital River Laboratory Animal Technology Co., Ltd. (Beijing, China). The mice were housed in an SPF environment and provided with standard rodent chow and water ad libitum.

### Dual-luciferase reporter gene assay

The interaction between TP63 (NCBI Reference Sequence: NM_003722.5) and hsa-miR-125b-5p was predicted using RNAhybrid software [19]. Sequence analysis revealed that nucleotides 2666–2693 of the TP63 3′ untranslated region (UTR) constituted a potential binding site for miR-125b-5p. To validate this prediction, luciferase reporter constructs were generated containing either the wild-type (TP63-WT) or a mutated (TP63-MUT) TP63 3′ UTR fragment (RiboBio, China) encompassing this target site. The fluorescence reporter in the vector was encoded by the Renilla luciferase (Rluc) gene, and these constructs were cloned downstream of the Rluc gene. For functional validation, HEK-293T cells were co-transfected with either the TP63-WT or TP63-MUT reporter plasmid and either miR-125b-5p mimics or mimic negative control (mimic NC; both from RiboBio, China). After 48 h of transfection, the Glo® Luciferase Assay System (Promega, U.S.A.) was used to lyse the cells, and luciferase activity was measured using a Glomax20/20 Luminometer (Promega, U.S.A.).

### Mice tongue tissues collection and the normal culture

The mice were anesthesized with CO_2_ and euthanized by cervical dislocation, and the tongue tissues were immediately harvested on a super-clean bench (Thermo, U.S.A.) and rinsed several times with sterile phosphate-buffered saline (PBS; KeyGEN, China) containing 5% penicillin/streptomycin (Gibco, U.S.A.). The tissues were subsequently cultured *in vitro* in a normal culture medium consisting of DMEM/F12 medium (Gibco, U.S.A.) supplemented with 10% fetal bovine serum (Gibco, U.S.A.) and 1% penicillin/streptomycin, in a humidified atmosphere of 5% CO_2_ at 37°C. The culture medium was replaced every 48 h with fresh medium. After culturing for the designated time periods, the tissues were collected for observation and hematoxylin and eosin (H&E) staining.

### Dsg3-monoclonal antibody treatment of mice tongue tissues

The collection and processing of mouse tongue tissues were performed as described above. After tissues were transferred to the normal culture medium and incubated in humidified atmosphere of 5% CO_2_ at 37°C, 0.5% Dsg3-monoclonal antibody (mAb) (ABclonal, China) was added to the culture medium, and the tissues were incubated for 2–48 h. The tissues were then collected for H&E staining and immunohistochemistry (IHC).

### miRNA transfection and Dsg3-mAb treatment of mice tongue tissues

The collection and processing of mouse tongue tissues were performed as described above. After the tissues were transferred to the normal culture medium and incubated in a humidified atmosphere of 5% CO_2_ at 37°C, they were cultured *in vitro* for 1 h to allow tissue stabilization. The miR-125b-5p mimic (sequence 5′-UCCCUGAGACCCUAACUUGUGA-3′, with no specific chemical modifications) or mimic NC (both from RiboBio, China) was diluted in DMEM/F12 medium, mixed thoroughly, and then combined with HighGene plus Transfection reagent (ABclonal, China) to form the transfection complex following the manufacturer’s instructions. Subsequently, the transfection complex of miR-125b-5p mimics or mimic NC was added into the culture medium for transfection, which was used at a final concentration of 50 nM. After 6 h, half of the culture medium was replaced with fresh normal culture medium, and after 24 h, the entire medium was removed. The tissues were then cultured in the normal culture medium with or without 0.5% Dsg3-mAb for 6 h. A normal culture group was cultured in the normal culture medium for the same duration. Samples were washed three times with PBS to remove extracellular mimics before lysis. The tissues were then collected for quantitative real-time reverse transcription PCR, H&E staining, IHC and observation of desmosome structure by transmission electron microscopy (TEM).

### H&E staining and IHC analyses

Mouse tongue tissue samples were embedded in paraffin. After sectioning, H&E staining was performed conventionally, and IHC was conducted for Dsg3, P63, and PERP (ABclonal, China). The stained sections were observed and photographed using a microscope (Nikon ECLIPSE 80i, Japan). For quantitative analysis of protein levels, IHC images were analyzed using ImageJ 1.8.0 software to calculate the average optical density (AOD) using the following equation: AOD = integrated optical density/area.

### RNA isolation and qRT-PCR

Total RNA was extracted from mouse tongue tissues using the mirVana™ RNA Isolation Kit (Thermo Fisher Scientific, U.S.A.) according to the manufacturer's specifications. cDNA was synthesized from the extracted RNA using the TransScript® First-Strand cDNA Synthesis SuperMix (TransGen Biotech, China). Real-time PCR was performed using 10 μL of PCR reaction mixture prepared with the PerfectStart™ Green qPCR SuperMix (TransGen Biotech, China) on a Roche LightCycler® 480II (Roche, Germany). The primer sequences were designed in-house and synthesized by TsingKe Biotech (Beijing, China) (Table 1). The relative expression levels of target mRNAs were calculated using the 2^−ΔΔCt^ method [20].

**Table 1:** Primer sequences used for qPCR or miRNA detection

Genes/miRNAs	Primer type	Sequences (5′ → 3′)
P63	Forward Reverse	ATTCCCTCCAACACAGATTAC GCTTCTTCAGTTCGGTGGAATA
PERP	Forward Reverse	GCACTGGCTGCCATATTC CCATAGGCCCAGTTATAGATGT
Gapdh	Forward Reverse	GCAAGGACACTGAGCAAGA GGATGGAAATTGTGAGGGAG
hsa-miR-125b-5p	Forward	CTGAGACCCTAACTTGTGAAA

### TEM observation of desmosomes structure

After mouse tongue tissues were excised, they were immediately transfected with miR-125b-5p mimic or mimic NC for 24 h and then treated with Dsg3-mAb for 6 h. A normal culture group was incubated in the normal culture medium for the same duration. In addition, a group of freshly excised normal tissues (without *in vitro* culture) was collected as the untreated control. All tissues were subsequently processed for TEM observation of desmosome structures. After collection, 2 × 1 × 1 mm epithelial tissue blocks were immediately dissected from the tongue tissues and placed in 1.25% glutaraldehyde fixation solution. After the postfixation with 2% osmium tetroxide, dehydration through an acetone series, and embedding, ultrathin sections were cut using an ultramicrotome, and the sections were double-stained with uranyl acetate and lead citrate. The desmosome structure was then observed using a H7000 TEM (HITACHI, Japan). Ten randomly selected visual fields per sample were observed at 10,000× and 15,000× magnification. Photomicrographs were captured at 15,000× magnification. The interdesmosomal width and the depth of intracellular keratin filament insertion were quantified using ImageJ 1.8.0 software.

### Statistical analysis

Quantified results are expressed as the mean ± standard deviation (SD). Statistical analyses were performed using GraphPad Prism version 6.01 (GraphPad Software, U.S.A.) or SPSS version 20.0 (IBM, U.S.A.). The Shapiro–Wilk test and Levene’s test were used to check the assumptions of normality and homogeneity of variance. Since the data failed to comply with normality and homogeneity of variances assumptions, the two-tailed Mann–Whitney *U* test was used for comparisons between two independent groups, and the Kruskal–Wallis test was used for comparisons among three or more groups. The sample size (*n*) for each experiment is indicated in the respective figure legends. Effect sizes (*r*) for Mann–Whitney *U* tests were calculated as $r=\left|Z\right|/\sqrt{N}$, where *Z* is the *Z*-score derived from the U-statistic and *N* is the total number of observations. Effect sizes *r* > 0.5 is considered a large effect. Differences with *P*<0.05 were considered statistically significant.

## Results

### MiR-125b-5p negatively regulated the expression of P63

To determine whether miR-125b-5p negatively regulates P63, we performed a dual-luciferase reporter assay. Positions 2666–2693 in the P63 3′ UTR were predicted to bind to miR-125b-5p. Co-transfection of the miR-125b-5p mimic with the TP63-WT reporter construct significantly decreased relative luciferase activity in HEK-293T cells compared with the mimic NC (*P<*0.001). In contrast, mutation of the putative binding site abolished this inhibitory effect (Figure 1). These results suggest that miR-125b-5p specifically targets the 3′ UTR of P63 and negatively regulates its expression.

**Figure 1:**
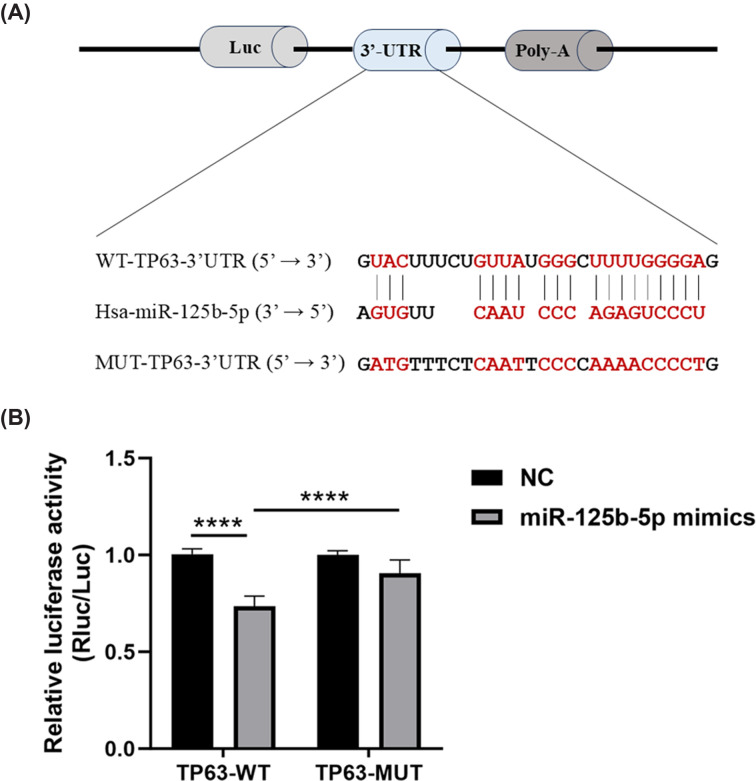
MiR-125b-5p directly targets the 3′ UTR of P63 (**A**) Predicted interaction between hsa-miR-125b-5p and the 3′ UTR of TP63-WT (NM_003722.5) at genomic position ∼2666. The miR-125b-5p (3′→5′) is shown aligned with the complementary target TP63 sequence (5′→3′). Vertical lines indicate base pairing. The miRNA seed region (nucleotides 2–8 5′→3′: UCCCUGA) is highlighted for functional relevance. Minimum free energy (Δ*G*) of the interaction: −25.0 kcal/mol. (**B**) Relative luciferase activity of the co-transfection of miR-125b-5p mimic with TP63-WT reporter construct was significantly lower compared with mimic NC, whereas this suppression was abrogated in the TP63-MUT group. Data are expressed as mean ± SD (*n* = 3 independent experiments). ****P<0.0001.

### Mouse tongue tissues remain stable after 96 h of normal *in vitro* culture

Mouse tongue tissues were cultured *in vitro* for 24, 48, 72, and 96 h, during which they exhibited a pink color and smooth, homogeneous appearance. The culture medium remained clear, with no visible tissue debris or impurities.

After 24 h of *in vitro* culture, the histopathological features of the tongue tissues appeared normal. H&E staining revealed intact epithelial and lamina propria architecture with well-preserved cell morphology and organized arrangement. The basement membrane appeared intact, and basal layer cells exhibited normal features. After 48–96 h of culture, exfoliation of the epithelial layer and edematous changes in individual epithelial cells were observed (Figure 2).

**Figure 2:**
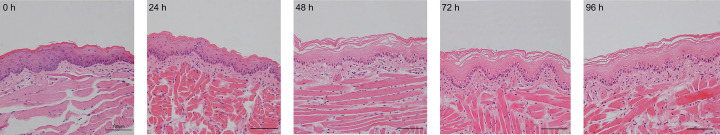
Histological changes in mouse tongue tissues after 24–96 h of normal culture No obvious histological changes were observed after 24 h of *in vitro* culture. Exfoliation of the epithelial layer and edematous changes in individual epithelial cells were observed at 48, 72, and 96 h of culture.

### Mouse tongue tissues develop PV-like pathological changes after 6 h of 0.5% Dsg3-mAb treatment

To establish histopathological and immunopathological features resembling PV for subsequent experiments, we treated mouse tongue tissues with 0.5% Dsg3-mAb for 6 h. H&E staining showed that the mouse tongue tissues maintained a normal mucosal structure after 2 h of culture, with no histopathologic changes compared with the 0 h (uncultured) control tissues. When the culture duration reached 6 h, intraepithelial destruction and vesicle formation began to appear. After 12, 24, and 48 h of culture, the tissues exhibited prominent intraepithelial vesicles (Figure 3A). Following 6 h of incubation with 0.5% Dsg3-mAb, IHC staining demonstrated that Dsg3 expression was significantly decreased in mouse tongue tissues compared with normal culture conditions (Figure 3B). Therefore, a 6 h treatment with 0.5% Dsg3-mAb was selected for subsequent experiments.

**Figure 3:**
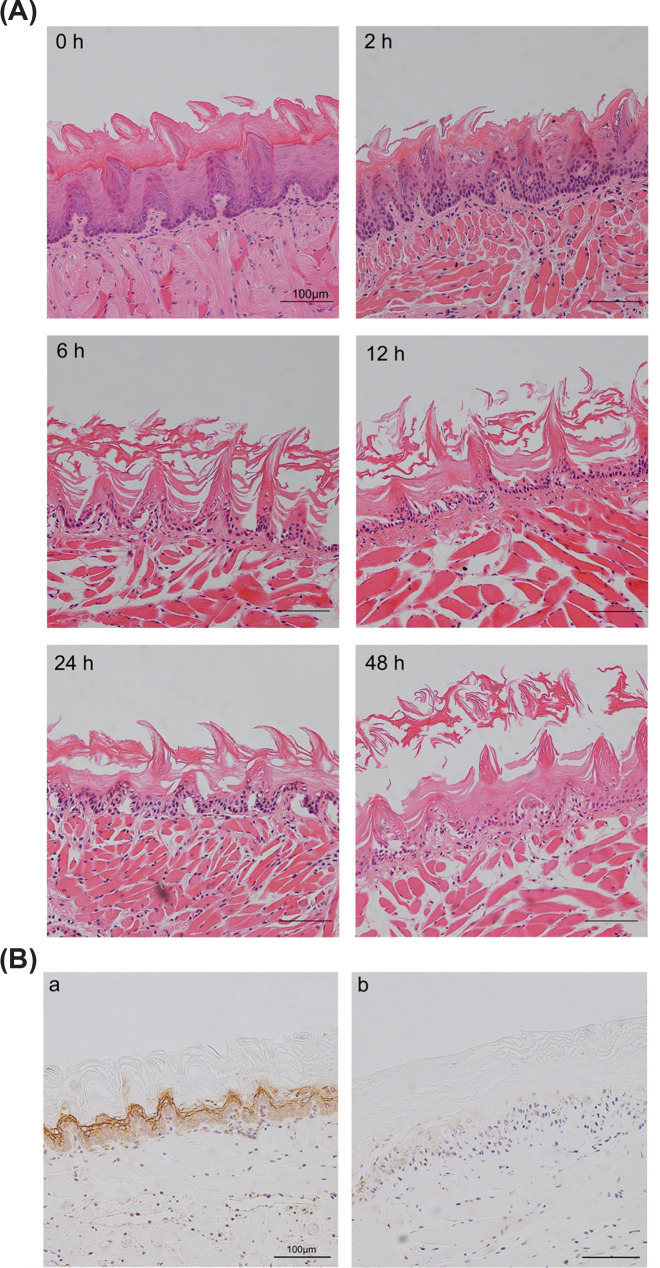
H&E staining and IHC image of mouse tongue tissues treated with 0.5% Dsg3-mAb (**A**) H&E staining of mouse tongue tissues treated with 0.5% Dsg3-mAb for 2, 6, 12, 24, and 48 h showed intraepithelial destruction at 6, 12, 24, and 48 h. (**B**) **(a)** IHC staining showed normal Dsg3 expression in the epithelial layer of mouse tongue tissue with 6 h of normal culture. **(b)** Dsg3 expression was significantly reduced in the epithelial layer of mouse tongue tissue following 6 h of treatment with 0.5% Dsg3-mAb, compared with normal culture.

### MiR-125b-5p promotes down-regulation of P63 and PERP in mouse tongue tissues treated with Dsg3-mAb

After 24 h of miR-125b-5p mimic transfection, miR-125b-5p expression in mouse tongue tissues was significantly higher than that in the mimic NC and normal culture groups, indicating successful transfection (Figure 4).

**Figure 4:**
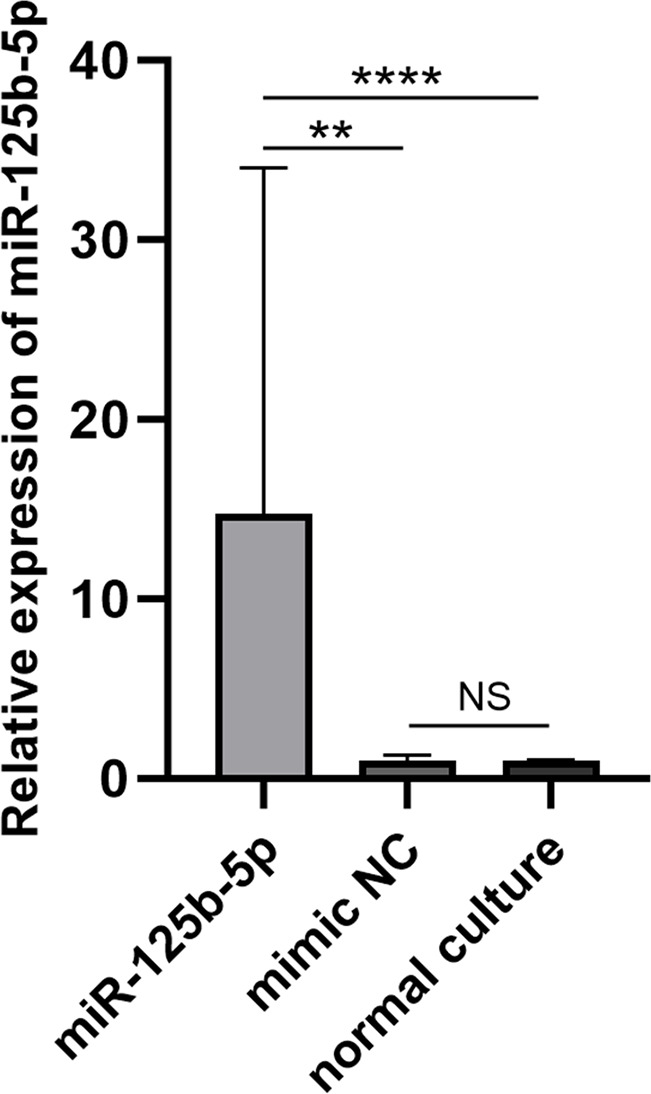
MiR-125-5p expression levels in miR-125b-5p mimic, mimic NC, and normal culture groups MiR-125b-5p mimic transfection significantly increased miR-125-5p expression compared with the mimic NC-transfected and normal cultured groups. Data are presented as mean ± SD (*n* = 10). **P<0.01 ****P<0.0001, NS = Not Significant.

Following transfection of the miR-125b-5p mimic or mimic NC in the presence of 0.5% Dsg3-mAb or normal culture medium, H&E staining, qRT-PCR, and IHC assays were performed to assess histopathological changes and the expression of P63 and PERP in mouse tongue tissues. H&E staining revealed noticeable intraepithelial blisters and acantholysis in tissues transfected with the miR-125b-5p mimic or mimic NC and treated with Dsg3-mAb, with remaining residual epithelial cells. Under normal culture conditions, tissues transfected with the miR-125b-5p mimic or mimic NC exhibited largely normal histological features (Figure 5A).

**Figure 5:**
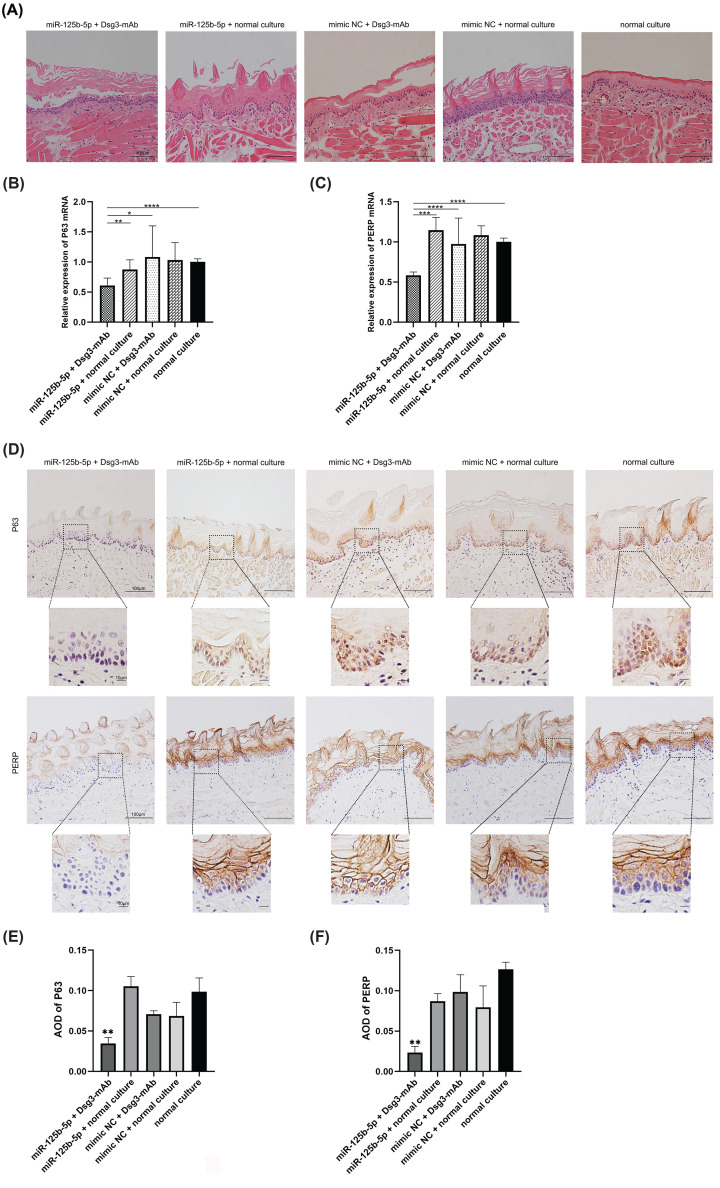
Effects of miR-125b-5p overexpression and Dsg3-mAb treatment on histology, mRNA, and protein levels of P63 and PERP in mouse tongue tissues (**A**) H&E staining showed that mouse tongue tissues transfected with miR-125b-5p mimic or mimic NC and treated with Dsg3-mAb developed intraepithelial blisters and acantholysis. In the absence of Dsg3-mAb (including miR-125b-5p mimic-transfected and mimic NC-transfected groups) and in normally cultured tissues, histological features remained largely normal. qRT-PCR analysis revealed significant reductions in P63 (**B**) and PERP (**C**) mRNA levels in tissues transfected with miR-125b-5p and treated with Dsg3-mAb. (**D**) IHC staining showed negligible positive signals for P63 and PERP in miR-125b-5p-overexpressed tissues treated with Dsg3-mAb. Semi-quantitative analysis of the IHC revealed significant reductions in P63 (**E**) and PERP (**F**) protein levels in miR-125b-5p-overexpressed tissues treated with Dsg3-mAb. Data are presented as mean ± SD (*n* = 10). *P<0.05, **P<0.01, ***P<0.001, ****P<0.0001.

qRT-PCR was performed to measure mRNA levels P63 and PERP. P63 expression in miR-125b-5p transfected tongue tissues cultured with Dsg3-mAb was significantly lower than that in mimic NC-transfected tissues under the same condition (*P*<0.05, *r* = 0.59). In miR-125b-5p-overexpressing tissues, P63 expression was significantly lower in the Dsg3-mAb-treated group than in the normal culture group (*P*<0.01, *r* = 0.69) (Figure 5B). PERP level in miR-125b-5p-overexpressed tongue tissues treated with Dsg3-mAb were significantly lower than those in mimic NC-transfected tissues with Dsg3-mAb treatment (*P*<0.0001, *r* = 0.85) and in tissues exposed to normal culture alone (*P*<0.001, *r* = 0.78). No significant differences in PERP levels were observed between miR-125b-5p-overexpressed tissues under normal culture and the tissues with normal culture (Figure 5C).

IHC analysis was performed to assess P63 and PERP expression. No positive staining of P63 or PERP was observed in tongue tissues with miR-125b-5p-overexpression and Dsg3-mAb treatment. In contrast, miR-125b-5p-overexpressed tissues under normal culture, mimic NC-transfected tissues with Dsg3-mAb treatment, and the blank control showed positive P63 and PERP expression in the epithelium (Figure 5D). Semi-quantitative analysis of IHC staining showed a significant reduction in P63 and PERP levels in the miR-125b-5p-overexpressed tissues treated with Dsg3-mAb (Figure 5E,F). These findings indicate that miR-125b-5p overexpression contributes to the down-regulation of P63 and PERP in tissues treated with Dsg3-mAb.

### MiR-125b-5p overexpression disrupts desmosome structure in Dsg3-mAb-treated mouse tongue tissues

As shown in Figure 6A, the red and the yellow bidirectional arrows represent the interdesmosomal width and the depth of intracellular keratin filament insertion, respectively. In miR-125b-5p-overexpressed tissues treated with Dsg3-mAb, the desmosome structure was disrupted and the cell membrane appeared blurred. Compared with the mimic NC-transfected tissues treated with Dsg3-mAb, the interdesmosomal width was significantly wider in miR-125b-5p-overexpressed tissues treated with Dsg3-mAb (44.80 ± 9.84 nm versus 35.48 ± 2.11 nm, *P*<0.0001, *r* = 0.85). The interdesmosomal width in these two groups was significantly higher than that in the normal cultured and untreated normal tissues. No significant differences were observed between the tissues with normal culture (28.60 ± 2.15 nm) and normal tissues (29.22 ± 2.86 nm) (Figure 6B). Similarly, the depth of intracellular keratin filaments insertion was significantly lower in miR-125b-5p-overexpressed tissues treated with Dsg3-mAb than in mimic NC-transfected tissues treated with Dsg3-mAb (39.85 ± 9.84 nm versus 54.17 ± 2.11 nm, *P<*0.01, *r* = 0.73), and was also lower than that in the normal cultured and normal tissues. (Figure 6C). These findings suggested that miR-125b-5p overexpression may contribute to desmosome disruption in mouse tongue tissues treated with Dsg3-mAb.

**Figure 6:**
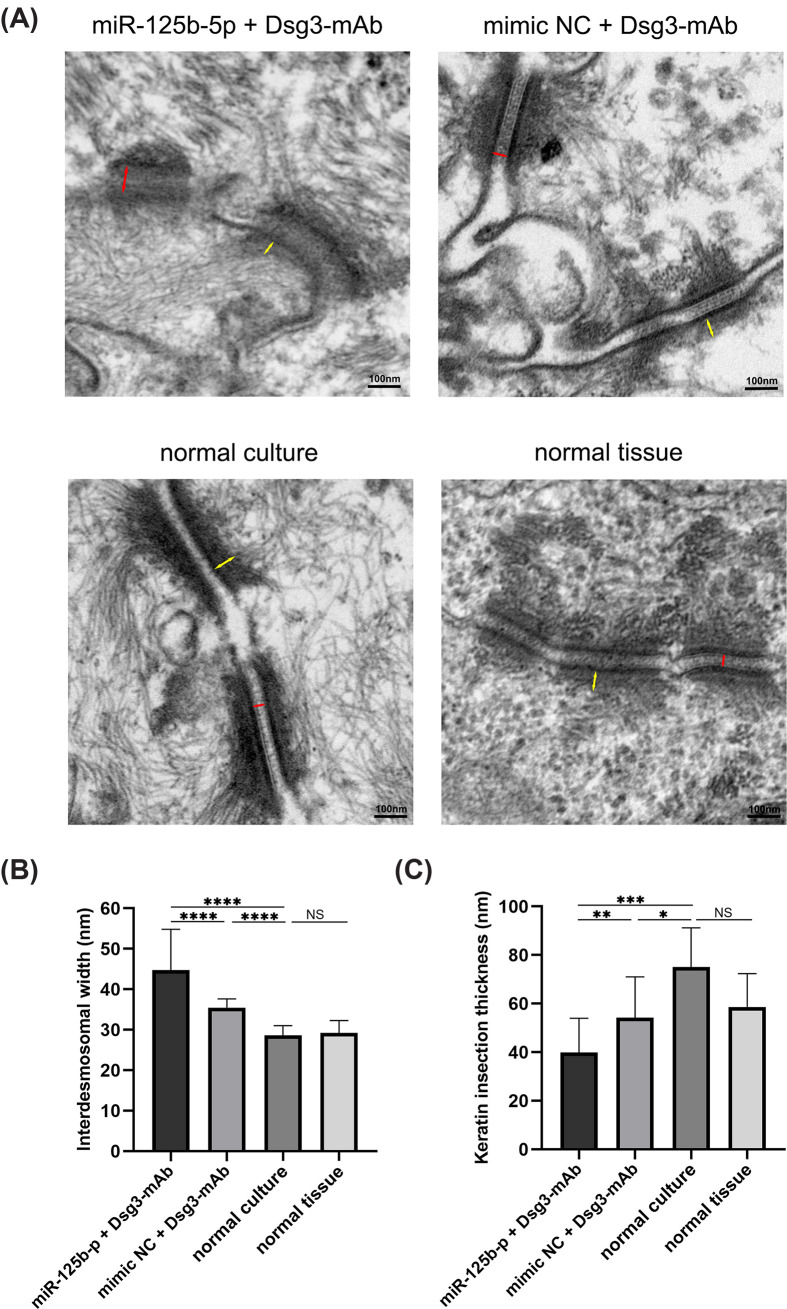
Effects of miR-125b-5p overexpression and Dsg3-mAb treatment on desmosome structures in mouse tongue tissues (**A**) TEM images of mouse tongue tissues from the following groups: miR-125b-5p overexpression with Dsg3-mAb treatment, mimic NC transfection with Dsg3-mAb treatment, normal culture, and normal tissues which were just *ex vivo* and untreated. Desmosome structures were disrupted, and cell membranes appeared blurred in the miR-125b-5p-overexpressed tissues treated with Dsg3-mAb, and the interdesmosomal width showed widening compared with the other three groups. Red bidirectional arrows represent the interdesmosomal width. Yellow bidirectional arrows represent the depth of intracellular keratin filament insertion. (**B**) The interdesmosomal width was significantly increased in miR-125b-5p-overexpressed tissues treated with Dsg3-mAb. (**C**) The depth of intracellular keratin filaments insertion was significantly reduced in miR-125b-5p-overexpressed tissues treated with Dsg3-mAb. Data are expressed as mean ± SD (*n* = 10). *P<0.05, **P<0.01, ***P<0.001, ****P<0.0001, NS = Not Significant.

## Discussion

Historically, mouse and human epidermal keratinocytes have been cultured *in vitro* and incubated with PV IgG to identify the pathogenic autoantibodies in PV [21,22]. Currently, epidermal keratinocyte cultures with elevated calcium continue to serve as a widely used *in vitro* model to investigate the molecular mechanisms driving loss of cell-cell adhesion in PV. The keratinocyte culture system, allowing for precise molecular manipulation through application of inhibitors/activators, miRNA transfection, and RNA interference, serves as a readily manipulable *in vitro* model ideally suited to dissecting the pathogenic mechanisms of cellular pathways or molecules underlying PV [23,24]. Nevertheless, monolayer keratinocytes do not recapitulate the three-dimensional architecture of epithelium and are unable to establish proper intercellular adhesion complexes, resulting in non-physiological changes. Additionally, the cultured cells are incapable of replicating immune interactions that play a critical role in pemphigus pathogenesis [25]. The *in vivo* murine models have also been employed in PV studies. Passive transfer models are generated through the administration of pathogenic antibodies into neonatal or adult C57Bl/6J mice, resulting in PV-like clinical and histopathological manifestations in the mice [26]. Alternatively, established active transfer mouse models involve the transfer of autoreactive lymphocytes into mice, inducing a complex autoantibody-driven clinical phenotype via transgenic approaches [27,28]. Although the mouse models recapitulate certain aspects of PV disease, they inherently present some disadvantages. Neonatal mice are inadequate for the detection of oral lesions, while adult passive transfer models require large quantities of pathogenic antibodies to induce disease. These active transfer models require two distinct mouse strains and exhibit low disease penetrance without immunization (17%) [29]. Furthermore, they demand highly specialized personnel and resource-intensive protocols [30]. In our prior study on autoimmune diseases, we cultured mouse submandibular and parotid gland tissues *in vitro*, and following incubation with interleukin-17, the tight junction expression and distribution were significantly altered [31]. Therefore, to obtain a reproducible and stable intact oral mucosa model comprising all layers and immune cells, we implemented the *in vitro* culture of mouse tongue tissues and treatment with Dsg3 mAbs in the present study. After 96 h of *in vitro* culture, mouse tongue tissues maintain a stable appearance and largely normal histopathological features. Following treatment with Dsg3-mAb for 6 h, mouse tongue tissues exhibit PV-like pathological changes. The immunostaining of Dsg3 showed significant reduction intensity, which perfectly mirrors the profound disruption of desmosomal infrastructure observed in Dsg-3mAb-treated tissues in Figure 6. This correlation between molecular signal loss and physical structural dissolution firmly demonstrates that the anti-Dsg3 antibody treatment triggered a genuine pathological depletion and junctional cleavage, rather than a technical artifact such as epitope masking. This represents a novel approach to establishing a reproducible, stable, accessible, and easy-to-operate *in vitro* model that reflects disease features of PV to the greatest extent.

Our previous study found that miR-125b-5p was significantly up-regulated in the serum of PV patients; however, its role in the pathogenesis of PV remains unclear [13]. MiR-125b-5p is involved in various pathophysiological processes and modulates the expression of genes associated with biological functions such as cell morphology, cellular movement, cell death, connective tissue organization, cardiovascular function, and the cell cycle [14]. In Hailey–Hailey disease (HHD), a subtype of pemphigus that is similar with PV in the clinical and histopathological features, miR-125b-5p was found to be overexpressed in HHD lesional keratinocytes when compared with their non-lesional keratinocytes [17]. P63, a member of the p53 family of transcription factors, plays a crucial role in epidermal development, differentiation, and maintenance. Mutations in P63 and its downstream genes are associated with various ectodermal dysplasias, which severely impair the normal structure and function of the skin and mucosa [16]. The P63 protein is a key regulator of epithelial development, differentiation, and intercellular adhesion. Reduced P63 levels impair cell adhesion, leading to the disruption of cell adhesion components such as Dsgs, integrins, laminins, cadherins, basement membrane complexes, and collagens [15,16,32]. Previous studies have shown that miR-125b-5p inhibits P63 expression by binding to its mRNA and blocking its translation, as confirmed by our dual-luciferase reporter assay [17,33,34]. Given that ΔNp63 is the most abundant isoform in the epidermis and is the primary transcriptional regulator of desmosomal proteins, it is highly likely that the down-regulation of the ΔNp63 isoform is the driving force behind the observed desmosome disruption [16]. Compared with the mimic NC-transfected and Dsg3-mAb treated group, P63 expression was significantly lower in miR-125b-5p-overexpressed and Dsg3-mAb-treated tongue tissues, suggesting that miR-125b-5p overexpression contributes to P63 degradation in PV, supporting an interaction between miR-125b-5p and P63 in PV disease.

PERP, a tetraspanin membrane protein, is a crucial molecule for maintaining normal desmosome assembly and epithelial barrier integrity and is directly regulated by the transcription factor P63 [18,35,36]. Studies have shown that P63 mutations can cause disorders such as Ankyloblepharon-ectodermal dysplasia-cleft lip/palate (AEC) syndrome, which are characterized by defects in desmosomal adhesion and epithelial integrity, further highlighting the critical role of P63 in regulating PERP and maintaining epithelial structure [15,37]. Additionally, reduced PERP expression is associated with impaired cell-cell adhesion and epithelial homeostasis in certain epithelial cancers [38,39]. Furthermore, in autoimmune bullous disease, Perp-knockout mice developed oral mucosal and skin blisters with histopathological features of PV, including intraepithelial blistering and acantholysis, underscoring the importance of PERP in cell adhesion and its crucial role in bullous diseases [18]. In the present study, the PERP mRNA levels were significantly reduced in the tongue tissues overexpressing miR-125b-5p and treated with Dsg3-mAb. At the protein level, the expression of both P63 and PERP was significantly reduced in miR-125b-5p-overexpressed and Dsg3-mAb treated tongue tissues, suggesting miR-125b-5p overexpression contributes to the down-regulation of both proteins in PV. In the Dsg3-mAb-treated tissues, miR-125b-5p overexpression contributed to the destruction of desmosome structures, as evidenced by the widening of interdesmosomal width and reduced depth of intracellular keratin filament insertion. Our findings suggest that miR-125b-5p negatively regulates P63, leading to reduced PERP expression and contributing to desmosome integrity impairment. These results indicate that miR-125b-5p promotes desmosome damage in PV by inhibiting P63 and subsequently down-regulating PERP. Accordingly, these observations support the existence of a potential miR-125b-5p/P63/PERP signaling pathway underlying desmosome abnormalities in PV. Given the pleiotropic nature of miR-125b-5p in regulating diverse cellular processes, it is likely that the miR-125b-5p/P63/PERP axis represents one facet of a broader regulatory network of miR-125b-5p in PV pathogenesis, the full complexity of which warrants further investigation. A proposed molecular mechanism of miR-125b-5p in PV is shown in Figure 7, created by FigDraw.

**Figure 7:**
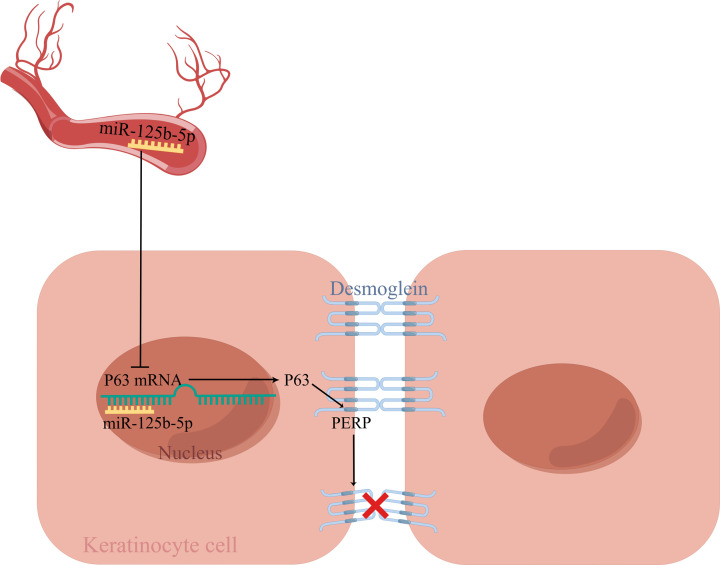
Proposed molecular mechanism by which miR-125b-5p overexpression contributes to desmosome disruption through inhibition of P63 and PERP (by FigDraw)

In summary, the present study investigated changes in P63 and PERP levels and their impact on desmosome structure in mouse tongue tissues following miR-125b-5p overexpression and Dsg3-mAb treatment, providing a new molecular mechanism involving the miR-125b-5p/P63/PERP pathway in PV pathogenesis. PV patients with high miR-125b-5p expression may exhibit additional desmosome damage beyond Dsg, including PERP, potentially leading to more severe disease and a worse prognosis. Furthermore, targeting and inhibiting miR-125b-5p may represent a promising adjunctive therapeutic strategy for PV patients with elevated miR-125b-5p levels. However, the present study has some limitations. First, the pathogenic mechanism of the miR-125b-5p/P63/PERP pathway in PV onset and progression needs further investigation. Future studies will incorporate functional rescue experiments to firmly establish mechanistic causality, alongside *in vivo* assays with expanded experimental sample numbers. The pleiotropic regulatory network mediated by miR-125b-5p in PV involves complex mechanisms that warrant deeper exploration. Second, further studies are needed to evaluate miR-125b-5p expression in PV patients at different disease stages and to explore its correlation during disease prognosis. Additionally, future studies will utilize multiple reference genes to ensure the highest standards of methodological stringency.

## Conclusions

MiR-125b-5p negatively regulates P63 and subsequently down-regulates PERP, leading to the disruption of desmosome structures and epithelial integrity. This miRNA is involved in the pathogenesis of PV, and its overexpression exacerbates tissue damage, suggesting that it may serve as a potential therapeutic target for PV, although its precise mechanisms and therapeutic value warrant further investigation.

## Data Availability

The datasets used and analyzed during the present study are available from the corresponding author on reasonable request.

## Abbreviations

**3**′ **UTR**: 3′ untranslated region
**AEC syndrome**: Ankyloblepharon-ectodermal dysplasia-cleft lip/palate syndrome
**AOD**: average optical density
**Dsg**: desmoglein
FBS: fetal bovine serum
**H&E**: hematoxylin and eosin
**HHD**: Hailey–Hailey disease
**IHC**: immunohistochemistry
**mAb**: monoclonal antibody
**mimic NC**: mimic negative control
**miRNAs**: microRNAs
**PBS**: phosphate-buffered saline
**PERP**: p53-targeted apoptosis effector related to PMP-22
**PV**: pemphigus vulgaris
**qRT-PCR**: quantitative real-time reverse transcription PCR
**Rluc**: Renilla luciferase
**SD**: standard deviation
**SPF**: specific pathogen-free
**TEM**: transmission electron microscopy
**TP63-MUT**: TP63 mutated-type
**TP63-WT**: TP63 wild-type

## References

[1] Amagai M., Tanikawa A., Shimizu T., Hashimoto T., Ikeda S., Kurosawa M. et al. (2014) Japanese guidelines for the management of pemphigus. J. Dermatol. 41, 471–486 10.1111/1346-8138.12486 24909210

[2] Schmidt E., Kasperkiewicz M. and Joly P. (2019) Pemphigus. Lancet 394, 882–894 10.1016/S0140-6736(19)31778-7 31498102

[3] Buonavoglia A., Leone P., Dammacco R., Di Lernia G., Petruzzi M., Bonamonte D. et al. (2019) Pemphigus and mucous membrane pemphigoid: An update from diagnosis to therapy. Autoimmun. Rev. 18, 349–358 10.1016/j.autrev.2019.02.005 30738958

[4] Harman K.E., Brown D., Exton L.S., Groves R.W., Hampton P.J., Mohd Mustapa M.F. et al. (2017) British Association of Dermatologists’ guidelines for the management of pemphigus vulgaris 2017. Br. J. Dermatol. 177, 1170–1201 10.1111/bjd.15930 29192996

[5] Kasperkiewicz M., Ellebrecht C.T., Takahashi H., Yamagami J., Zillikens D., Payne A.S. et al. (2017) Pemphigus. Nat. Rev. Dis. Primers 3, 17026 10.1038/nrdp.2017.26 28492232 PMC5901732

[6] Langan S.M., Smeeth L., Hubbard R., Fleming K.M., Smith C.J.P. and West J. (2008) Bullous pemphigoid and pemphigus vulgaris–incidence and mortality in the UK: population based cohort study. BMJ 337, a180 10.1136/bmj.a180 18614511 PMC2483869

[7] Wertenteil S., Garg A., Strunk A. and Alloo A. (2019) Prevalence estimates for pemphigus in the United States: a sex- and age-adjusted population analysis. JAMA Dermatol. 155, 627–629 10.1001/jamadermatol.2018.5954 30892569 PMC6506879

[8] Slack F.J. and Chinnaiyan A.M. (2019) The role of non-coding RNAs in oncology. Cell 179, 1033–1055 10.1016/j.cell.2019.10.017 31730848 PMC7347159

[9] Long H., Wang X., Chen Y., Wang L., Zhao M. and Lu Q. (2018) Dysregulation of microRNAs in autoimmune diseases: pathogenesis, biomarkers and potential therapeutic targets. Cancer Lett. 428, 90–103 10.1016/j.canlet.2018.04.016 29680223

[10] Miao C., Wang X., Zhou W. and Huang J. (2021) The emerging roles of exosomes in autoimmune diseases, with special emphasis on microRNAs in exosomes. Pharmacol. Res. 169, 105680 10.1016/j.phrs.2021.105680 34010670

[11] Evangelatos G., Fragoulis G.E., Koulouri V. and Lambrou G.I. (2019) MicroRNAs in rheumatoid arthritis: from pathogenesis to clinical impact. Autoimmun. Rev. 18, 102391 10.1016/j.autrev.2019.102391 31520804

[12] Singh R.P., Hahn B.H. and Bischoff D.S. (2022) Identification and contribution of inflammation-induced novel microRNA in the pathogenesis of systemic lupus erythematosus. Front. Immunol. 13, 848149 10.3389/fimmu.2022.848149 35444657 PMC9013931

[13] He W., Xing Y., Li C., Zhou P., Hu X., Hua H. et al. (2022) Identification of six microRNAs as potential biomarkers for pemphigus vulgaris: from diagnosis to pathogenesis. Diagnostics (Basel) 12, 3058 10.3390/diagnostics12123058 36553065 PMC9776764

[14] Nagpal V., Rai R., Place A.T., Murphy S.B., Verma S.K., Ghosh A.K. et al. (2016) MiR-125b is critical for fibroblast-to-myofibroblast transition and cardiac fibrosis. Circulation 133, 291–301 10.1161/CIRCULATIONAHA.115.018174 26585673 PMC5446084

[15] Ferone G., Mollo M.R., Thomason H.A., Antonini D., Zhou H., Ambrosio R. et al. (2013) p63 control of desmosome gene expression and adhesion is compromised in AEC syndrome. Hum. Mol. Genet. 22, 531–543 10.1093/hmg/dds464 23108156 PMC3542863

[16] Koster M.I. (2010) p63 in skin development and ectodermal dysplasias. J. Invest. Dermatol. 130, 2352–2358 10.1038/jid.2010.119 20445549 PMC2919658

[17] Manca S., Magrelli A., Cialfi S., Lefort K., Ambra R., Alimandi M. et al. (2011) Oxidative stress activation of miR-125b is part of the molecular switch for Hailey–Hailey disease manifestation. Exp. Dermatol. 20, 932–937 10.1111/j.1600-0625.2011.01359.x 21913998

[18] Ihrie R.A., Marques M.R., Nguyen B.T., Horner J.S., Papazoglu C., Bronson R.T. et al. (2005) Perp is a p63-regulated gene essential for epithelial integrity. Cell 120, 843–856 10.1016/j.cell.2005.01.008 15797384

[19] Rehmsmeier M., Steffen P., Hochsmann M. and Giegerich R. (2004) Fast and effective prediction of microRNA/target duplexes. RNA 10, 1507–1517 10.1261/rna.5248604 15383676 PMC1370637

[20] Livak K.J. and Schmittgen T.D. (2001) Analysis of relative gene expression data using real-time quantitative PCR and the 2(-Delta Delta C(T)) method. Methods 25, 402–408 10.1006/meth.2001.1262 11846609

[21] Amagai M., Klaus-Kovtun V. and Stanley J.R. (1991) Autoantibodies against a novel epithelial cadherin in pemphigus vulgaris, a disease of cell adhesion. Cell 67, 869–877 10.1016/0092-8674(91)90360-B 1720352

[22] Stanley J.R. and Yuspa S.H. (1983) Specific epidermal protein markers are modulated during calcium-induced terminal differentiation. J. Cell Biol. 96, 1809–1814 10.1083/jcb.96.6.1809 6343404 PMC2112456

[23] Hofrichter M., Dworschak J., Emtenani S., Langenhan J., Weiß F., Komorowski L. et al. (2018) Immunoadsorption of desmoglein-3-specific IgG abolishes the blister-inducing capacity of pemphigus vulgaris IgG in neonatal mice. Front. Immunol. 9, 1935 10.3389/fimmu.2018.01935 30233569 PMC6130267

[24] Mao X., Choi E.J. and Payne A.S. (2009) Disruption of desmosome assembly by monovalent human pemphigus vulgaris monoclonal antibodies. J. Invest. Dermatol. 129, 908–918 10.1038/jid.2008.339 19037235 PMC2743719

[25] Van Der Wier G., Pas H.H. and Jonkman M.F. (2010) Experimental human cell and tissue models of pemphigus. Dermatol. Res. Pract. 2010, 143871 10.1155/2010/143871 20585596 PMC2877615

[26] Schulze K., Galichet A., Sayar B.S., Scothern A., Howald D., Zymann H. et al. (2012) An adult passive transfer mouse model to study desmoglein 3 signaling in pemphigus vulgaris. J. Invest. Dermatol. 132, 346–355 10.1038/jid.2011.299 21956125 PMC3258361

[27] Koch P.J., Mahoney M.G., Ishikawa H., Pulkkinen L., Uitto J., Shultz L. et al. (1997) Targeted disruption of the pemphigus vulgaris antigen (desmoglein 3) gene in mice causes loss of keratinocyte cell adhesion with a phenotype similar to pemphigus vulgaris. J. Cell Biol. 137, 1091–1102 10.1083/jcb.137.5.1091 9166409 PMC2136216

[28] Ohyama M., Amagai M., Tsunoda K., Ota T., Koyasu S., Hata J. et al. (2002) Immunologic and histopathologic characterization of an active disease mouse model for pemphigus vulgaris. J. Invest. Dermatol. 118, 199–204 10.1046/j.0022-202x.2001.01643.x 11851895

[29] Hartmann V., Hariton W.V., Rahimi S., Hammers C.M., Ludwig R.J., Müller E.J. et al. (2023) The human skin organ culture model as an optimal complementary tool for murine pemphigus models. Lab. Anim. 57, 381–395 10.1177/00236772221145647 36647613

[30] Emtenani S., Hertl M., Schmidt E. and Hudemann C. (2023) Mouse models of pemphigus: valuable tools to investigate pathomechanisms and novel therapeutic interventions. Front. Immunol. 14, 1169947 10.3389/fimmu.2023.1169947 37180099 PMC10172572

[31] Zhang L.W., Cong X., Zhang Y., Wei T., Su Y.C., Serrão A.C. et al. (2016) Interleukin-17 impairs salivary tight junction integrity in Sjögren’s syndrome. J. Dent. Res. 95, 784–792 10.1177/0022034516634647 26933138

[32] Carroll D.K., Carroll J.S., Leong C.O., Cheng F., Brown M., Mills A.A. et al. (2006) p63 regulates an adhesion programme and cell survival in epithelial cells. Nat. Cell Biol. 8, 551–561 10.1038/ncb1420 16715076

[33] Xu N., Brodin P., Wei T., Meisgen F., Eidsmo L., Nagy N. et al. (2011) MiR-125b, a microRNA downregulated in psoriasis, modulates keratinocyte proliferation by targeting FGFR2. J. Invest. Dermatol. 131, 1521–1529 10.1038/jid.2011.55 21412257

[34] Odar K., Boštjančič E., Gale N., Glavač D. and Zidar N. (2012) Differential expression of microRNAs miR-21, miR-31, miR-203, miR-125a-5p and miR-125b and proteins PTEN and p63 in verrucous carcinoma of the head and neck. Histopathology 61, 257–265 10.1111/j.1365-2559.2012.04242.x 22690848

[35] Ihrie R.A. and Attardi L.D. (2005) A new Perp in the lineup: linking p63 and desmosomal adhesion. Cell Cycle 4, 873–876 10.4161/cc.4.7.1836 15970683

[36] Jheon A.H., Mostowfi P., Snead M.L., Ihrie R.A., Sone E., Pramparo T. et al. (2011) PERP regulates enamel formation via effects on cell-cell adhesion and gene expression. J. Cell Sci. 124, 745–754 10.1242/jcs.078071 21285247 PMC3039019

[37] Clements S.E., Techanukul T., Lai-Cheong J.E., Mee J.B., South A.P., Pourreyron C. et al. (2012) Mutations in AEC syndrome skin reveal a role for p63 in basement membrane adhesion, skin barrier integrity and hair follicle biology. Br. J. Dermatol. 167, 134–144 10.1111/j.1365-2133.2012.10888.x 22329826

[38] Dusek R.L., Bascom J.L., Vogel H., Baron S., Borowsky A.D., Bissell M.J. et al. (2012) Deficiency of the p53/p63 target Perp alters mammary gland homeostasis and promotes cancer. Breast Cancer Res. 14, R65 10.1186/bcr3171 22515648 PMC3446400

[39] Khan I.A., Yoo B.H., Masson O., Baron S., Corkery D., Dellaire G. et al. (2016) ErbB2-dependent downregulation of a pro-apoptotic protein Perp is required for oncogenic transformation of breast epithelial cells. Oncogene 35, 5759–5769 10.1038/onc.2016.109 27109096

